# Aptasensors for Point-of-Care Detection of Small Molecules

**DOI:** 10.3390/bios10090108

**Published:** 2020-08-26

**Authors:** Marc Prante, Ester Segal, Thomas Scheper, Janina Bahnemann, Johanna Walter

**Affiliations:** 1 Institute of Technical Chemistry, Leibniz Universität Hannover, Callinstr. 5, 30167 Hannover, Germany; prante@iftc.uni-hannover.de (M.P.); scheper@iftc.uni-hannover.de (T.S.); jbahnemann@iftc.uni-hannover.de (J.B.); 2 Department of Biotechnology and Food Engineering, Technion Israel Institute of Technology, Technion City, Haifa 3200003, Israel; esegal@technion.ac.il

**Keywords:** aptamer, small molecules, healthcare, point-of-care testing, POCT

## Abstract

Aptamers, a group of nucleic acids which can specifically bind to a target molecule, have drawn extensive interest over the past few decades. For analytics, aptamers represent a viable alternative to gold-standard antibodies due to their oligonucleic nature combined with advantageous properties, including higher stability in harsh environments and longer shelf-life. Indeed, over the last decade, aptamers have been used in numerous bioanalytical assays and in various point-of-care testing (POCT) platforms. The latter allows for rapid on-site testing and can be performed outside a laboratory by unskilled labor. Aptamer technology for POCT is not limited just to medical diagnostics; it can be used for a range of applications, including environmental monitoring and quality control. In this review, we critically examine the use of aptamers in POCT with an emphasis on their advantages and limitations. We also examine the recent success of aptasensor technology and how these findings pave the way for the analysis of small molecules in POCT and other health-related applications. Finally, the current major limitations of aptamers are discussed, and possible approaches for overcoming these challenges are presented.

## 1. Introduction

Aptamers, which are short nucleic acid sequences, were described in 1990 for the first time and proposed to be a promising alternative to antibodies [1,2]. In the following years, aptamers were extensively tested and established in a variety of applications. Yet, after all these years, only a handful of aptamers are commercially used (and approved) in diagnostics and therapeutics. As for the latter, the most prominent therapeutic aptamer is Macugen by Pfizer, which was approved by the FDA in 2004, for treating neovascular age-related macular degeneration (AMD) [3]. Additional anti-angiogenic aptamers for AMD treatment (Zimura, Fovista and Pegnivacogin) are currently in advanced stages of clinical trials [4,5], along with a few more aptamers for other renal diseases [6]. Aptamers are widely developed and successfully applied for numerous diagnostic applications and yet their translation into commercial use in general and point-of-care testing (POCT) is still in its infancy. In this review, we provide a thorough overview on the potential of aptamers for flexible diagnostics. We highlight the challenges in the use of aptamers for diagnostics in general and POCT applications. We chose to focus on small-molecule health markers, as their detection by conventional antibody-based approaches can be limited due to a couple of factors.

## 2. Small Molecule Targets

Small molecules, which are characterized by a low molecular weight (<900 Daltons) are organic compounds which may regulate biological processes [7]. There are naturally occurring low molecular weight compounds such as food-contaminants. Many of these contaminants are produced by filamentous fungi and exhibit a wide range of potential harmful effects on health. The most abundant and critical mycotoxins for human health are aflatoxins (produced by the *Aspergillus* species), ochratoxins (produced by *Penicillium* and *Aspergillus* species) and Fusarium toxins (produced by over 50 species of *Fusarium*) [8,9]. Alongside these naturally occurring compounds, many small molecules are anthropogenic. Among these are polychlorinated biphenyls (PCBs), which have been widely used in industrial applications. In the 1980s, the use of PCBs was mostly banned in all countries, since the compounds exhibit moderate toxic potential in animals and humans and cannot be degraded naturally [10,11]. There are also small molecules which act as pesticides and are used in agriculture. In this context the most prominent compound is Glyphosate which was extensively discussed in the media over the past year due to its potential carcinogenic effects [12,13]. 

Many pharmaceuticals are defined as small molecules. For drug application this could be extremely attractive, since small molecules can pass the blood–brain barrier due to their size [14,15,16]. Modern medicine is unimaginable without pharmaceuticals and they are used all around the world. They are ubiquitous and can also be found in the environment as a pollutant through human excretion. The most analyzed pharmaceuticals in the environment are antibiotics, followed by analgesics and hormones. In 713 analyzed water samples originating from all around the world, 631 were found to contain these pharmaceutical compounds [16]. Moreover, small molecules often play important roles in regulatory pathways in the human body. Vitamins, hormones, messenger molecules and cofactors are different groups of small molecules which regulate the metabolism. 

The ever-growing interest in monitoring these compounds in the environment—as well as in the human body—led to a rising interest in the development of a variety of sensors for the detection of small molecules. Due to their ubiquitous nature and important functions, small-molecule targets are of high interest and extensive research has been carried out in this area in the past years. In the last ten years, 17,912 research articles dealing with small molecules were published (Web of Science, keyword “small molecules”, 07/31/20). Commonly, small molecules are detected via chromatographic techniques, such as high-pressure liquid chromatography (HPLC) and gas chromatography (GC). However, these methods are often expensive, require trained technicians and are time consuming. A promising alternative for detection and monitoring of small molecules is the use of biosensors.

## 3. Biosensors

Biosensors are bioanalytical devices containing a biological element (such as, cells, antibodies, enzymes or oligonucleotides [17]) which selectively reacts/binds with the target of interest. The resulting biological recognition events are converted into a measurable signal by the transducer. The first use of a biosensor, reported by Clark and Lyons in 1962, was for the detection and quantitation of glucose concentration in blood [18]. Glucose oxidase was immobilized on a semi-permeable membrane, which encased an oxygen electrode, and a decrease in the measured oxygen concentration oxygen concentration was directly correlated to the glucose concentration [18]. 

In general, there are four groups of transduction methods which are usually used in biosensors.

- Optical
- Piezoelectric
- Calorimetric
- Electrochemical

In optical transduction methods, recognition binding events are converted into measurable changes in various optical properties, such as fluorescence, refractive index and diffraction. Piezoelectric transducers are based on changes of the molecular weight upon target binding. Since enzyme catalyzed reactions are usually exothermic, these changes in heat can be monitored by calorimetric transducers. Finally, electrochemical transducers, such as the mentioned Clarke-biosensor, induce changes of current, impedance or ion concentrations [19].

## 4. Aptasensors

**Figure 1:**
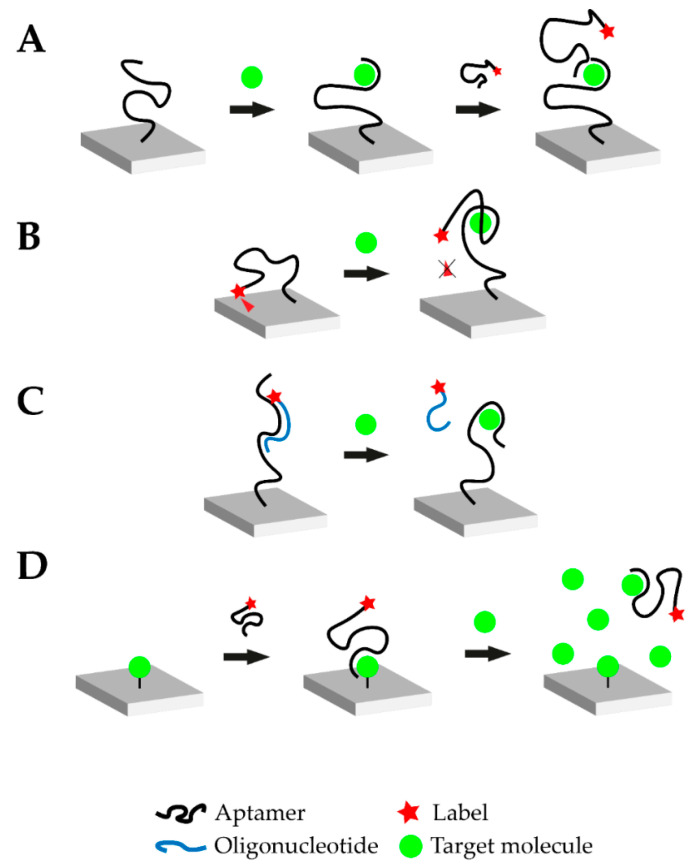
Overview of the different possible operational modes of aptasensors. (**A**) Sandwich or sandwich-like mode: One aptamer is immobilized on a surface and the target molecule is added. Upon addition, the aptamer-target complex is formed. Afterwards, another labeled aptamer against the respective target is introduced which binds to a different epitope of the target molecule. Transduction is carried out via the labeled aptamer. (**B**) Target-induced structure switching (TISS) mode: The labeled aptamer is immobilized on a surface. In this example, a fluorescence molecule is linked to the aptamer and brought near a quenching surface. Upon target addition, the aptamer structure changes and the fluorescence molecule is no longer quenched. In this case, transduction is carried out via fluorescence measurement. (**C**) Target-induced dissociation (TID) mode: A complementary, labeled oligonucleotide hybridizes with the immobilized aptamer. Upon target addition, the aptamer-target complex is formed and the oligonucleotide is displaced. After washing steps, the respective signal decreases and transduction can be carried out. (**D**) Competitive replacement (CR) mode: In this example, the target molecule is immobilized on the surface. The labeled aptamer is added and forms the aptamer-target complex. When the target molecule is added in excess, the aptamer preferably binds to the free molecule and is removed from the immobilized target molecule. This change can then be monitored.

In 1990, three laboratories independently announced the establishment of a new in vitro selection method for nucleic acid sequences which bind their target in a highly selective manner. This technique, termed as SELEX (systematic evolution of ligands by exponential enrichment), resulted in the discovery of aptamers [1,2]. During the SELEX process, a library of random oligonucleotide sequences with up to 10^18^ individual nucleic acid sequences is exposed to the desired target. A small percentage of the library’s sequences binds to the target and subsequently separates. The latter are amplified via polymerase chain reaction (PCR) and the selection process is typically repeated for 8–15 rounds [20]. To increase selectivity, counter selection may be performed (addition of molecular structures similar to the target) and the sequences binding to the non-target structures are removed. Due to their stringent selection process, aptamers offer a feasible alternative to antibodies and they offer several prominent advantages. Aptamers are chemically synthesized at high reproducibility and low cost compared to antibodies and they are stable over a wide range of temperature and pH, as well as in organic solvents [20,21,22]. These significant advantages, along with their small size and the ease of their immobilization and regeneration, have led to extensive research on aptamer-based biosensors [23]. The latter are termed aptasensors and the oligonucleic acid is commonly immobilized on the biosensor surface. Immobilization drastically improves handling of the biosensor and the nucleic acid can be regenerated more easily [24]. It is possible to design competitive assays through the application of complementary strands, which bind the aptamer, offering new possibilities for biosensing in general and small molecules in particular. The oligonucleotide nature of aptamers and complementary strands offer the possibility to implement amplification and improve aptasensor sensitivity. Figure 1 schematically illustrates four typical modes of action of aptasensors: Sandwich or sandwich-like mode, target-induced structure switching mode, target-induced dissociation mode and finally competitive replacement mode [25].

### 4.1. Sandwich or Sandwich-Like Mode

Similar to the concept of enzyme-linked immunosorbent (ELISA) assays, sandwich-like detection methods have been established for aptamers. The oligonucleotide is immobilized on a surface and binds its target, where a second aptamer binds a different binding site of the target to form a “sandwich” structure (Figure 1A). This mode can be realized with two aptamers directed against different epitopes of the target or in mixed approaches using an aptamer and an antibody. Therefore, it is possible to create aptamer-target-antibody hybrids. Such hybrid-systems were successfully demonstrated to detect cardiovascular biomarkers, such as troponin, but were not yet tested with small molecules [26,27]. The small molecule progesterone could also be detected in a sandwich-like lateral flow assay with a final LOD of 5 nM using gold-immobilized aptamers [28]. Small molecules do not offer many structural recognition elements which consequently limit the applicability of the sandwich-like strategy. Moreover, due to the small size of the target, steric hinderance between the two binding aptamers is likely to happen. Yet, it should be noted that when antibodies are employed this difficulty may intensify due to their larger size and limited flexibility when binding epitopes.

### 4.2. Target-Induced Structure Switching (TISS) Mode

Target-induced structure switching (TISS) mode uses the aptamer’s ability to fold into a defined 3D-structure upon target binding. When the target molecule is introduced, the aptamer undergoes structural rearrangements, as illustrated in Figure 1B. The aptasensor could be designed to either measure changes in size of the aptamer upon target binding, the position of binding moieties or structural stability. However, when detecting small molecules these structural changes can be marginal and challenging to be implemented. Yet, many TISS mode aptasensor for the detection of small molecules are described in literature and several examples are listed in Table 1. Several aptasensors for the detection of ATP using the TISS mode were described in literature and were successfully tested in aptamer binding buffer [29,30,31]. Moreover, extensive research was carried out for cocaine aptasensors which were able to detect up to pM cocaine concentrations in serum [32]. 

### 4.3. Target-Induced Dissociation (TID) Mode 

Like the sandwich-like and TISS modes, the target-induced displacement of complementary oligonucleotides approach (TID), illustrated in Figure 1C, also relies on the structural properties of the aptamer. However, the change of the aptamer conformation is not monitored directly, but the displacement of a complementary oligonucleotide is detected. This oligonucleotide is usually designed to bind in the aptamer-target binding site or in a place, where drastic structural changes occur upon target binding. Upon target binding, the complementary oligonucleotide is displaced, which enables the detection of this signal change for transduction (Figure 1C). Usually, the aptamer is immobilized on the biosensor surface and the oligonucleotide is added. For transduction purposes, the complementary oligonucleotide can be modified at its 5’ or 3’-terminus. One possible modification is the addition of a Cy5 or Cy3 molecule to monitor the change in fluorescence intensity upon target addition. This would represent an optical aptasensor. Lee et al. demonstrated the ability of a TID-based AU-NP aptasensor to monitor the small molecule hydroyvitamin D3 (25(OH)D_3_) in the micromolar range [33]. These findings could be verified and further extended by the introduction of Cy5-labelled complementary oligonucleotides [34].

### 4.4. Competitive Replacement (CR) Mode

In the competitive replacement mode, free target molecule competes with immobilized target molecule. Since binding of the free target molecule is preferred, the labelled aptamer is not able to bind to the immobilized target in the presence of the target (Figure 1D). A competitive aptasensor for the detection of chloramphenicol (CHL) could be developed based upon this principle which was able to detect CHL with a LOD of 451 pM. CHL was immobilized on a surface and biotin-labelled CHL-aptamer was introduced. When no free CHL was available, the CHL-aptamer bound the immobilized CHL and transduction was performed via streptavidin-modified gold nanoparticles (AuNPs). However, when free CHL was available the CHL-aptamer formed a complex with it and no sandwich-like structure on the surface could be formed [35]. Another aptasensor with the competitive replacement mode could be tested successfully for the detection of the small molecule neomycin B. In this setup, the target antibiotic is immobilized on a surface and then bound by the aptamer. Upon addition of higher neomycin B concentrations, the aptamer is displaced and binds to the free neomycin B. This change is then transduced with faradaic impedance spectroscopy (FIS). This aptasensor was capable of measuring the small molecule with a LOD in the µM range [36]. Another competitive aptasensor for the detection of the small molecule aflatoxin B1 (AFB1) could be developed. The anti-AFB1-aptamer was fused with the anti-thrombin-aptamer. The aptasensor surface was coated with immobilized AFB1. When no free AFB1 was present, the aptamer-fusion-complex was able to bind to the sensor surface. The anti-thrombin-aptamer then captured thrombin which cleaves a peptide substrate. This cleavage was then used as the transduction method. However, when free AFB1 is present, the aptamer-fusion-complex does not bind to the immobilized AFB1 and the substrate cannot be cleaved by thrombin after washing steps. The aptasensor was able to detect the mycotoxin with a LOD of 0.5 nM [37].

### 4.5. Applicability of the Different Aptasensor Modes for the Detection of Small Molecules

We compiled a table with a selection of aptasensors for the detection of small molecules with their respective operational modes such as competitive replacement (CR) mode, TISS, TID and sandwich-like mode. For the selection please see Table 1. It is evident that the majority of the presented aptasensors operate with the target induced structure switching mode, followed by competitive replacement and target-induced dissociation. The least used operational mode is the sandwich-like mode. This could be due to the necessity of having two aptamers directed against two different epitopes of the target analyte. Small molecules offer limited structural motifs for detection purposes and therefore complicate the design of sandwich-like assays even more. The second least used operational mode is the target induced dissociation mode when operating aptasensors. The use of TID requires structural knowledge of the used aptamer to identify either the target binding site or areas of strong structural changes. Only with this knowledge or “trial-and-error” it is possible to design the complementary oligonucleotides so that they are displaced upon target binding. In contrast to the TID mode no in-depth knowledge of the aptamer structure is needed for the TISS mode to operate successfully. The only requirement for a successful TISS mode is a significant structural change of the aptamer upon target binding. When this is not the case, the TID mode offers an effective alternative. When the displacement of the complementary oligonucleotide cannot be monitored with the transduction method, it is possible to label the oligonucleotide and amplify the transduction process, e.g., for mass-sensitive transduction methods. Another option to amplify transduction processes is the use of a variety of nanomaterials which also enable higher signal-to-noise ratios (SNR). 

### 4.6. Integration of Nanomaterials

In recent years, nanotechnology was embedded in a variety of aptasensors to improve sensitivity and SNR. Nanomaterials can be incorporated in two manners: Nanoparticles as signalling elements and nanostructured surfaces as transducers or amplificators. For the former, aptasensors which incorporate gold nanoparticles (AuNPs) and quantum dots (QDs) were reported with promising sensitivity and signal amplification [46,47]. AuNP-based biosensors often operate with an optical transduction method since the aggregation of the nanoparticles upon aptamer-target binding leads to a wavelength shift from 520 nm to 650 nm. Assays can be designed in a sandwich-like mode with two aptamers against the respective target. These two aptamers are then immobilized on the AU-NP-surface and the AuNPs aggregate upon target addition, which leads to a wavelength shift [48]. Since AuNPs have quenching properties they are suitable for fluorometric aptasensors. Electrochemical transduction methods in AuNP-based aptasensors have also been reported. The large surface area and high redox activity of the gold nanoparticles lead to signal enhancement and increased biosensor sensitivity [46].

Semiconducting nanocrystals, also called quantum dots, are another nanomaterial capable of significantly improving biosensing sensitivity and signal amplitude. In recent studies, QDs were functionalized with aptamers. A quencher-labelled complementary oligonucleotide was added which binds to the aptamer when no target is present. Upon target binding, the oligonucleotide dissociates and the fluorescence signal increases [49].

Another promising nanomaterial for the development of aptasensors is porous silicon (PSi) [50,51]. The latter is a nanostructured porous material with unique optical properties and a large surface area, allowing for a greater number of aptamers to be immobilized. Most PSi-based aptasensors are optical, in which binding of the target molecules to the immobilized aptamers induce changes in the reflectivity of the porous transducer owing to changes in the average refractive index.

**Figure 2:**
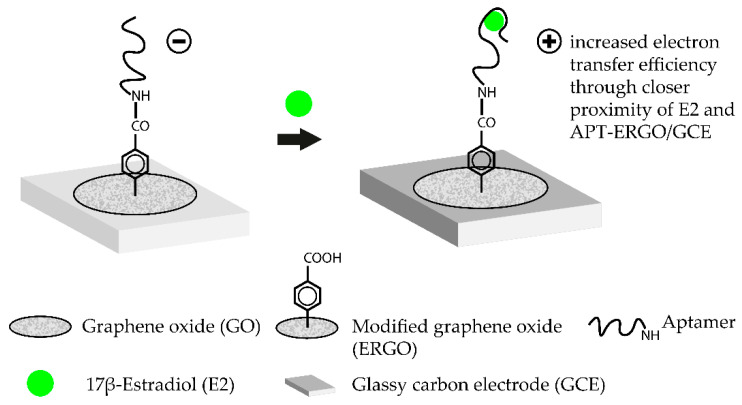
Working principle of a label-free aptasensor for the femtomolar detection of 17β-estradiol (E2) using graphene oxide as an amplification platform. To immobilize the estradiol-binding aptamer, a series of previous chemical modifications are necessary. Firstly, a glassy carbon electrode (GCE) is modified with a graphene oxide (GO) layer. The GO diazonium salts are then electrochemically reduced which leads to the ERGO/GCE hybrid surface. The COOH-groups on the ERGO surface are then conjugated with NH_2_-modified E2-aptamers via carbodiimide formation. Addition of the target molecule E2 leads to the formation of the E2-aptamer complex. In the complex, E2 is brought closer to the ERGO/GCE surface which leads to an increased electron transfer efficiency. Transduction is then carried out by measuring the change in current by using square wave voltammetry. The aptasensor operates in the TISS mode. (Adapted from [45]).

Such aptasensors were successfully demonstrated for label-free detection of various targets [52,53,54]. Nanostructured graphene materials, such as graphene oxide (GO), are another class of promising candidates for aptasensor surfaces. These materials offer excellent quenching properties. Additionally, single-stranded DNA (ssDNA) is easily adsorbed on the surface of the material and drastically improves the handling and generation of modified sensor surfaces [46]. Aptamer-coated GO surfaces were already successfully tested to detect the mycotoxin B1 in complex matrices such as beer or wine. The aptasensor operated in a TISS mode and exhibited a LOD of 0.05 ng·mL^−1^ [38]. A label-free approach for the femtomolar detection of the hormone 17β-estradiol with aptamer-coated GO surfaces could also be demonstrated [45]. The working principle of this TISS mode aptasensor is shown in Figure 2. Another novel aptasensing approach is the use of molecularly imprinted polymers (MIP) alongside aptamers to detect target molecules in an extremely sensitive matter. MIP are artificial recognition units which can be specifically tailored to detect certain compounds. Moreover, MIP are extremely stable and can be produced cheaply. By integrating aptamers and MIP, new diagnostic tools can be developed which combine both benefits in one device. Such hybrid-MIP approach was used to design an aptasensor for tetracycline which was able to detect the antibiotic with a LOD of 144 fM. The major advantage of this aptasensor was its stability. After storing the sensor for four weeks at ambient conditions, no drastic change in signal intensity upon biosensing could be monitored. Moreover, the hybrid-MIP could be used several times with consecutive denaturation and regeneration steps [55]. This biosensor illustrates one of the main advantages of aptasensors, since they can be used several times and can be regenerated easily. Antibodies are susceptible to permanent denaturation and are therefore heavily impacted by the solution in which the biosensor operates. In aptasensors, these problems do not arise [56].

In general, aptasensors offer a range of advantages compared to antibody-based biosensors. However, the already established and clinically proven immunoassays are difficult to compete with and moreover, existing aptasensors were only extensively tested for a small range of targets and usually in non-clinical environments (for example buffer). In the ever-growing field of medical diagnostics, aptasensors could fill an important gap between expensive and rather difficult to handle immunoassays and point of care (POCT) diagnostics. To fill this gap however, existing aptamers need to be optimized for monitoring these small molecules with an adequate sensitivity and selectivity. It could be shown that the sensitivity could be drastically improved when nanomaterials were incorporated in the aptasensor.

## 5. Comparison of Aptasensors and Immunosensors for Small Molecule Sensing

In the previous chapters we gave an overview of the possible operational modes of aptasensors and optimization possibilities using a variety of nanomaterials. Over the past decade, aptasensors were extensively used and optimized. This resulted in the construction of aptasensors which are superior to the well-established antibodies in terms of limit of detections. We compiled a table of these small molecule targets and compared the LOD of aptasensors with the LOD of immunosensors for these targets. The resulting Table 2 is shown below.

For the small molecules tetracycline, bisphenol A, ochratoxin A and estradiol the LODs of aptasensors and immunosensors are comparably low and enable sensitive biosensing possibilities. However, it is notable that the aptasensor for the detection of cocaine is superior (100 pM LOD) to the respective immunosensor (0.49 nM). To highlight the flexibility and possibilities of aptasensor development we describe the major accomplishments in cocaine aptasensing in the past years. This progress is notable, since this picomolar LOD was only achieved with a variety of aptasensor improvements over the past years. In 2006, the first aptasensor for cocaine detection was developed based on the target-induced structure switching (TISS) operational mode. The aptasensor exhibited a LOD of 10 µM which was too high for clinical applications [67]. This aptasensor was optimized using a different operational mode in 2009. In this biosensing approach, the sandwich-mode was used. Moreover, the cocaine-aptamer was split in two fragments, where one fragment was immobilized on the biosensor surface and the other one was labeled and freely available in solution. Upon cocaine addition, the equilibrium was shifted from the two separated aptamer fragments to the aptamer-target-complex and electrochemical transduction was carried out. This aptasensor was able to detect cocaine with a LOD of 1 µM [68]. The split-aptamer approach was extensively explored by other groups and refined using nanomaterials such as quantum dots (QD) and a variety of nanoparticles such as PtNPs and AuNPs [59,69,70]. In 2015, a nanocomposite aptasensor for the detection of cocaine was able to detect the compound with a final LOD of 100 pM. In this aptasensor, the aptamer is linked to AuNPs which are attached to a nanocomposite modified glassy carbon (GC) electrode. When no cocaine is present and the aptamer is partially unfolded, ferricyanide is brought close to the electrode surface. However, when cocaine is present, the negatively charged AuNPs approach the electrode surface, which results in inhibited electron transfer and the redox probe current decreases [59]. Even the most recent immunosensor for cocaine biosensing (0.49 nM [60]) is not able to compete with the LOD of 100 pM exhibited by the presented aptasensor. This drastic improvement of LOD over the past years highlights the high versatility of aptasensing technology. Once an aptamer against a certain target is selected, the biosensor design can be optimized to drastically improve the aptasensor performance. The different operational modes such as TID, TISS and Sandwich-like and the incorporation of nanomaterials offer optimization options to increase sensitivity. Of course, it is also possible to optimize immunosensors with nanomaterials and different operational modes. However, one major limiting factor in sensitivity is the immobilization density of the capture probe on the biosensor surface. Since the aptamer is drastically smaller than an antibody, higher immobilization densities are possible, leading to higher sensor efficiencies. The majority of targets shown in Table 2 are environmental contaminants, whereas Estradiol is the only small molecule relevant in health monitoring. In the following chapter we examine the recent advances in aptasensor technology for the detection of small molecules in health monitoring.

## 6. Current Status of Aptasensors for the Detection of Small Molecules in Health Monitoring

In recent years, due to increasing interest in small molecules, aptamers directed against these molecules were selected via SELEX. Since 2015, 1196 research articles or reviews have been written including the keyword “aptasensor” (Web of Science, 07/31/20). Moreover, a lot of these articles not only describe the selection process via SELEX, but also the development of an aptasensor for the detection of the selected target molecule. To give a brief overview of the currently available aptamers against important health markers, we used the “Richtlinie der Bundesärztekammer zur Qualitätssicherung” (guideline of the German medical association to maintain quality standards) (Rili-Bäk). This is a guideline for German laboratories to maintain quality standards. Major chemical industries such as Roche Diagnostics acknowledge this guideline and use it for their own POCT products. Based on the table in the Rili-Bäk with all relevant existing health markers, we removed analytes which are not defined as small molecules (<900 kDa) from the resulting table and checked whether an aptamer for the desired target was reported in literature. The resulting analytes and their indications as health markers are shown in Table 3.

As shown in Table 3, aptamers for many important hormones, which are analyzed in laboratory tests, are available in literature. Additionally, an aptamer for the detection of 25-hydroxyvitamin D_3_ is described in literature, which is another significant health marker since Vitamin D deficiency is a widespread illness. No aptamers were reported for aldosterone, bilirubin, uric acid and creatinine yet. In summary, the development of aptamers for targeting small molecules in health monitoring was mainly driven by the selection of aptamers against hormones. In addition, aptamers against other relevant small molecules such as cortisol and 25(OH)D_3_ were also selected. As hormones are clinically tested mostly by radioimmunometric methods which are costly and time extensive, these available aptamers offer the potential to develop aptasensors for point-of-care testing. However, with the use of aptasensors, some challenges arise both in general limitations of aptamers and in aptamer selection processes. In the following, we examine the major challenges in the detection of small molecules using aptasensors and how to solve those problems to make aptamers more attractive for POCT applications.

## 7. Challenges in the Detection of Small Molecules Using Aptasensors

Small molecules are one of the most extensively studied aptamer targets in literature. For example, the ATP aptamer was extensively studied in literature and thousands of articles were published using this aptamer. However, in the last 10 years only a handful of new aptamers against small molecules for health monitoring (besides the previously mentioned hormones) were selected and published. In this subsection we explore both, general limitations when using aptamers for the detection of small molecules and more specialized challenges which can occur in the selection process.

### 7.1. General Challenges

One of the major limitations of aptamers for the detection of small molecules is due to the small size of the target molecule. It was reported that the target molecular weight is proportional to the binding affinity of the aptamer in most cases [75,76]. This can be explained by the rather limited presence of functional groups in small molecules. These functional groups, however, are important for specific target recognition and target binding. In the theophylline binding aptamer it could be shown that hydrogen bonds are extremely important target recognition and binding [77]. Consequently, fewer functional groups lead to decreased specificity and binding affinity. This poses a problem for the development of new aptamers for small molecules since the target analytes are usually monitored in the nanomolar range (regarding health markers) and require high specificity. Patents for a variety of aptamer applications also significantly inhibited innovation processes so that researches did not decide to invest time and material in the discovery of new aptamers for small molecules [78].

### 7.2. SELEX-Related Challenges

**Figure 3:**
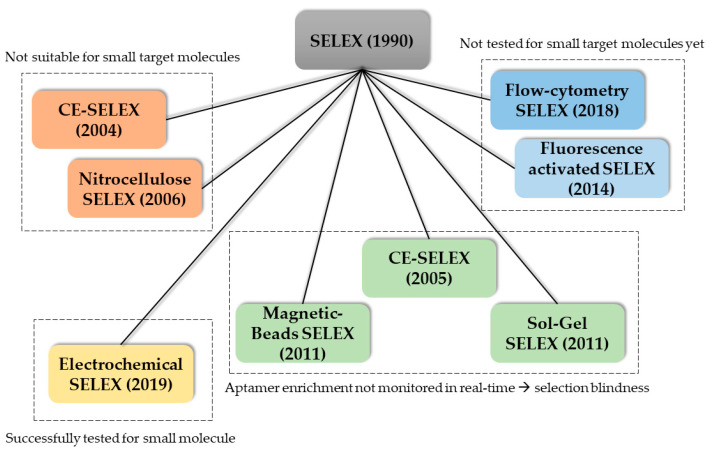
Overview of the reported SELEX methods used for the selection of new aptamers grouped based on their properties regarding applicability in small molecule selection and operational modes.

One main advantage of aptamers is the high degree of selectivity when the selection process (SELEX) is carried out optimally. This includes a sufficient amount of selections rounds (10–15) and counter selection rounds. Another important step in the SELEX process is the separation of the target-bound aptamer from the unbound oligonucleotides. Therefore, the target molecule is often immobilized on a surface. For protein targets, hydrophobic interactions with the surface may be sufficient for separation. For cell targets, centrifugation or fluorescence activated cell sorting (FACS) is used. When immobilizing small molecules however, these targets often lack functional groups needed for conjugation chemistry and therefore immobilization. Moreover, introduction of functional groups is problematic and alters the structure of the small molecule which could lead to false-positive binding. Additionally, aptamers of the selection library could falsely bind to the linker molecule. Moreover, introducing new functional groups is time consuming. The alternative, using the already existing functional groups further decreases the number of functional groups available for aptamer target recognition. Counter-selection steps should be carried out in SELEX to increase specificity and exclude cross-reactivity [79,80]. However, in an already time-consuming protocol these extra steps may drastically worsen the economics. Since the discovery of the SELEX process in 1990, a variety of new SELEX methods were reported in literature. For an overview of the existing SELEX methods, and their context in the use for the selection of small molecule aptamers, see Figure 3. The capillary electrophoresis (CE) SELEX and Nitrocellulose SELEX were successfully tested for the selection of new aptamers against proteins such as IgE and rhVEGF_165_ [81,82]. These new approaches offered faster selection rounds and a higher heterogeneity of the selection pools. However, both SELEX techniques are not suitable for the selection of small molecule aptamers since the target molecule needs to exhibit a certain size to be excluded in the separation steps. Microfluidic systems were also reported which incorporate magnetic-bead-based SELEX, CE-SELEX and Sol-Gel SELEX methods. These systems suffer from the lack of real-time monitoring of the aptamer enrichment. This results in more time-extensive selection processes and consequently higher experimental costs. Moreover, higher failure rates and selection blindness are reported. These circumstances make these methods (currently) not suitable for the selection of aptamers in the POCT context. A promising new method, called electrochemical SELEX could be successfully used to select and aptamer against the small molecule 11-Deoxycortisol. In this approach, the target molecule is immobilized on a gold electrode surface. Then, the interaction between the selection library and the target molecule can be monitored in real-time via voltammetric measurements. Compared with the conventional SELEX method, no labelling of the oligonucleotides is necessary [83]. Another promising SELEX method is the capture SELEX. Compared to the previously mentioned methods such as CE-SELEX, Sol-Gel SELEX and the classic SELEX, in this method the oligonucleotides are immobilized. Since the immobilization of small molecules inherits a variety of problems, immobilization of the oligonucleotide is usually preferred and drastically improve the selection process [84]. Moreover, the use of capture SELEX automatically leads to complimentary oligonucleotides. This facilitates the development of an aptasensor in TID mode since the complementary strand of ssDNA can be used directly in this aptasensor.

### 7.3. Challenges regarding K_d_ Determination

When new aptamers are selected via SELEX, an important classification number for characterization is the dissociation constant (K_d_). The lower the K_d_ the higher the affinity of the aptamer to the respective target. There are a variety of methods to measure K_d_ such as spectroscopy based methods, mass-sensitive methods and separation-based methods [85]. To determine the K_d_ either the aptamer or the target molecule is titrated against the other molecule. Since the aptamers are usually larger than the small molecule, this leads to low signal-to-noise ratios and binding events are difficult to detect. K_d_ determination methods can be classified in two groups: Immobilization of one binding partner (either aptamer or target) or immobilization-free methods. Immobilization-based methods include plasmon resonance (SPR), where one partner is immobilized and the K_d_ is measured. Immobilization-free methods include isothermal calorimetry (ITC), capillary electrophoresis (CE) and microscale thermophoresis (MST) [86]. Different methods were used to determine the K_d_ of the aptamer directed against the small molecule ochratoxine A (OTA). The results indicated a wide range of determined K_d_, ranging from 125 to 374 nM. The previously reported literature value of the OTA-binding aptamer was 200 nM [87]. High variability of experimentally detected dissociation constants shows one of the major problems in K_d_ determination. Since the K_d_ is an important classification factor for further aptasensor development, this could lead to a range of problems. However, this discrepancy between K_d_ measuring methods could also be due to their fundamentally different working methods. In one approach, the target or aptamer is immobilized, in the other method both binding partners are free in solution. Therefore, the K_d_ method should be chosen with the aptamer application in mind. For aptasensor approaches, where the aptamer is usually immobilized, methods such as SPR should be chosen to generate reliable K_d_ results. However, when looking for therapeutic aptamers for example, immobilization-free K_d_ determination methods such as ITC, MST or CE should be used. To approximate the factual K_d_, different methods (either immobilization-free or not) should be used, and a high number of replicates should be carried out. In recent years, a couple of promising new determination methods were reported. Isothermal Titration Calorimetry (ITC), which belongs to the calorimetric and label-free methods, was used successfully in the determination of K_d_ of aptamers against small molecules such as 25(OH)D_3_ and cocaine [33,88]. ITC offers fast results with a label-free experimental setup. However, high aptamer or target concentrations are needed to perform the ITC, therefore often resulting in costly experiments [89]. Another promising candidate for K_d_ determination is MicroScale Thermophoresis (MST). MST is based on the thermophoretic mobility of molecules in temperature gradients. The mobility of the molecule heavily depends on its size, hydration shell and charge, therefore when the aptamer-target complex is formed, a change in movement behavior can be monitored. This leads to the dissociation constant K_d_. Strong structural changes are needed to occur in the aptamer upon target binding to observe the K_d_ in the MST. The K_d_ determination of small molecule aptamers using MST could be shown in the 17β-estradiol-binding aptamer and the well-established ATP-aptamer. Additionally, the MST was carried out successfully for the characterization of the ochratoxin A-binding aptamer [90,91,92]. The main advantage of the MST is the low sample volume, fast generation of results and easy experimental layouts. The major downside is the need for labelled interaction partners. Usually the aptamer is fluorescently labelled, whereas it is also possible to label the target molecule. The labelling is usually carried out during chemical synthesis of the aptamer. As already mentioned, modifications and labels could alter the binding properties (and consequently the K_d_) and should be evaluated carefully. Automated microchip electrophoresis could be shown to be another promising method in K_d_ determination since it requires low reagent use and leads to fast K_d_ results [15].

## 8. Aptamers in Point-of-Care Testing (POCT) 

Point-of-care testing (POCT) is defined by the ability to instantly monitor health markers on-site (for example directly at the hospital) and get results quickly. The current workflow in the detection of health-markers is to take the biological sample at the hospital, send it to a central laboratory (when the hospital has no own laboratory) and get the results in a couple of days. However, this approach suffers from a couple of disadvantages. Firstly, experienced personnel are needed to analyze the biological sample in the laboratory, secondly, costly equipment is needed and finally, the samples need to be shipped to the testing facility. In the POCT approach, the biological samples are taken and analyzed at the same location. This is possible with simple-to-handle medical devices and rather cheap resources which are needed for the tests. When POCT is established, the health markers can be measured quickly, cheaply and without the need for experienced personnel. Moreover, it is possible to create a network of POCT institutions and continuously exchange data and experience. This would lead to a decentralized approach of health monitoring and would offer fast and reliable results for the customer, anywhere. However, the transition from the current approach to a POCT-approach is difficult. At the beginning of a POCT-transitional phase, the hospital suffers from extra effort and expense. Employees need to be trained to handle the POCT-devices, a central data point and managing software needs to be established and a solid quality management system needs to be established. The POCT should be a complementary approach to the central-laboratory, with the same quality-management and reproducibility. The resulting data needs to be comparable to the results of the central laboratories of the current approach. All these challenges need to be taken in account when an institution transitions from the current “central laboratory approach” to the POCT-approach. Aptasensor-based POCT incorporates all advantages of aptamers including long-term stability, aptamer regeneration and high sensitivity in the nanomolar range. In contrast to antibodies, aptamers in POCT devices could be more durable, cheaper and could withstand harsher environments, which could simplify prior sample preparation. Aptamers could especially excel in the detection of small molecules, which usually regulate important functions in the body and are relevant as health markers. One disadvantage of aptamers is their susceptibility to nucleases. However, this vulnerability to nucleases could be negligible attributable to the POCT approach. Firstly, measurements in POCT ideally should be carried out in a short period of time (e.g., 10 min), which limits the influence of nuclease activity. Secondly, the aptamers in POCT approaches will not be regenerated most of the time. In case they should be regenerated, it is possible to introduce aptamer modification to make the oligonucleotide more nuclease-resistant such as 3’-poly-dA tails or 3’-biotin caps for in vitro measurements [93,94]. Finally, aptamer immobilization itself makes the aptamers less prone for DNase activity. POCT systems should be easy to handle, cheap and fast. With the use of aptasensors as a base for new POCT systems, the first two mentioned points could be successfully established. The time aspect, however, needs to be evaluated thoroughly. Aptamer-target binding is a time-dependent step (until equilibrium is reached) with a potential optimum for POCT application. However, this spot needs to be evaluated carefully, since a too short incubation time may lead to insufficient binding and therefore unreliable results with low signal-to-noise ratios. This problem gets even worse when small molecules are used which lead to low signal-to-noise ratios by themselves. When designing aptasensor-based POCT applications, the incubation time is a crucial parameter which needs to be optimized previously—both for applicability and quality assurance. In the worst case, the desired sensitivity of the established aptasensor will not get used to its fullest extent. 

**Figure 4:**
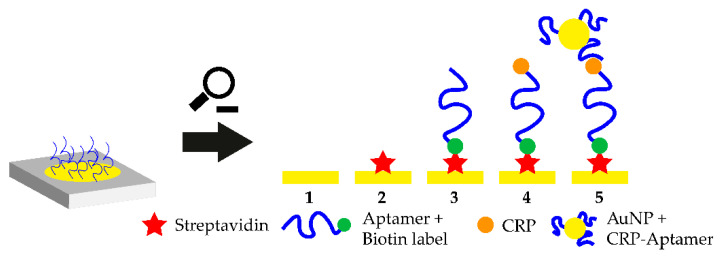
Close-up of the working principle of the smartphone-assisted sensor platform. Streptavidin is immobilized on a gold coated surface and the biotinylated CRP-aptamer is added. Biotin binds to the immobilized streptavidin and the aptamer binds to circulating CRP. Then, aptamer-modified gold nanoparticles (AuNPs) are added which bind to a different epitope on the CRP molecule. The shift in the surface plasmon resonance (SPR) wavelength is monitored with a smartphone-assisted device and CRP concentration can be measured.

A promising platform for POCT applications is the smartphone-assisted sensor. In 2020, 4.78 billion people on earth owned a smart and feature phone which makes smartphone-based POCT an extremely feasible approach which also enables at-home-testing of biological markers. In 2016, a smartphone-assisted aptasensor was reported. The sensor system consists of an optical waveguide, which guides the light from the smartphone through a SPR sensor to the output coupler. Then, the wavelength shift of the SPR is monitored. Upon target addition, the aptamer-target complex is formed and the wavelength shift is monitored. This smartphone-assisted sensor was successfully used for the detection of the C-reactive protein with a LOD of 12.46 nM (normal C-reactive protein (CRP) concentration in blood is 40 nM) [95]. The working principle of the sensor is shown in Figure 4. The working principle of this sandwich-like aptasensor is shown in Figure 3. The use of smartphones in POCT extends the idea of POCT so that self-testing of various health markers at home could be possible in the future. The only part which needs to be purchased in order to measure these analytes is the rather cheap polymer-based SPR sensor, while readout can be performed using the users own smartphone [96]. 

## 9. Conclusions and Future Prospects

In point-of-care testing, aptamers offer a wide range of advantages compared to antibodies when used as the biosensing platform. Aptamers have a long shelf-life and are more stable in rougher chemical conditions. Moreover, they can be produced in bulk in a much cheaper way than antibodies. Aptamers are especially suitable for the detection of small molecules since their small size results in large binding capacities when the aptamer is immobilized on the sensor surface. This is an advantage for the detection of small molecules, since more target analyte can be bound by the aptamer and transduced. Another advantage of aptamers for the detection of small molecules is their potential high specifity and a variety of different operational modes such as sandwich-like, TISS, TID and competitive mode. These modes make the biosensor development extremely flexible and new sensing approaches can be introduced easily. In POCT-systems aptamers pave the way for fast and efficient test kits such as lateral-flow assays or SPR-based biosensors with the incorporation of smartphones. However, the selection and characterization of aptamers for small molecules is the major bottleneck for the discovery of new aptamers against small molecules. The size of small molecules such as testosterone or other hormones leads to a variety of problems in the selection process and K_d_ determination. Problems include limited chemical groups needed for immobilization both in K_d_ determination and SELEX. Moreover, aptamers need to be selected more stringent, with more selection rounds, counter selection rounds and overhauled SELEX methods such as the electrochemical SELEX technique which makes nucleotide-labeling needless. Another bottleneck of aptasensing in the POCT area are inconsistent K_d_ determination methods. There are a variety of methods to determine the dissociation constant of an aptamer, however there are large deviations of the reported K_d_ between these methods. When characterizing aptamers for the POCT use, different measurement approaches should be used, and multiple measurements are necessary. Methods such as microscale thermophoresis and isothermal titration calorimetry are two of the most efficient K_d_ determination methods since the aptamer-target-complex is formed freely in solution and no immobilization is needed. Aptasensors for the detection of small molecules also need to leave proof-of-principle experiments behind and explore the real testing conditions. See Table 1 for a selection of recently reported aptasensor for the detection of small molecules. The majority of these aptasensors were tested in buffer systems and have not been tested for rather complex clinical samples such as human serum or environmental samples yet. These complex matrices confront the aptamer with a series of potential problems: Extremely low target concentration, cross-reactivity, other potential contaminants and nucleases. This environment could lead to low signal-to-noise ratios. An efficient starting point of SNR improvement is the optimization of the transduction method. Nanomaterials such as porous silicon (PSi), graphene oxide (GO) and gold nanoparticles (AuNPs) are recent advances in this field with immense potential for aptasensor improvement. These materials offer both efficient signal amplification methods and an aptamer immobilization surface which drastically improves the handling of the aptasensor. These possibilities and recent advances could lead to a dramatic increase in aptasensor research interest. Cocaine immunosensing is a good example which shows the potential of aptasensors in the current research environment. The first cocaine aptasensor was published in 2006 with moderate results. Since then, the sensor was drastically improved which led to an LOD increase of five-fold and successful biosensing serum in the femtomolar concentration range [32]. Until now, no superior antibody immunosensor could be developed for cocaine sensing and the aptasensor remains one of the most effective sensing approaches [97]. While aptasensors might still be in an early development state in the context of POCT, this example clearly demonstrates the high potential of aptasensors as well as the manifold options for their development and optimization.

## Figures and Tables

**Table 1:** Selection of aptasensors against a variety of small molecules with their operational modes, limits of detection (LOD) and sample composition.

Target	Mode	LOD	Sample Composition	Reference
Aflatoxin B1	TISS	pM	Beer/Wine	[38]
Aflatoxin B1	CR	nM	Buffer	[37]
Aflatoxin M1	TISS	nM	Spiked milk	[29]
Aflatoxin M1	CR	nM	Buffer	[30]
ATP	TISS	nM	Buffer	[39]
ATP	TISS	µM	Buffer	[31]
ATP	TISS	µM	Buffer	[40]
Ochratoxin A	TID	nM	Buffer	[41]
Ochratoxin A	TID	nM	Grape juice/serum	[42]
Chloramphenicol	CR	pM	Serum/milk	[35]
Cocaine	TISS	pM	Serum	[32]
Cocaine	TISS	nM	Buffer	[43]
Cocaine	TISS	nM	Buffer	[44]
Estradiol	TISS	fM	Buffer	[45]
Neomycin B	CR	µM	Buffer	[36]
Progesterone	Sandwich	nM	Buffer	[28]
25-Hydroxyvitamin D3	TID	µM	Serum	[33]

**Table 2:** Selection of superior aptasensors for the detection of small molecules compared with the most efficient immunosensor alternative based on limit of detection (LOD) values.

Target	LOD_Aptasensor_	LOD_Immunosensor_	Ref._Aptasensor_	Ref._Immunosensor_
Tetracycline	5 pM	13 pM	[57]	[58]
Cocaine	100 pM	0.49 nM	[59]	[60]
Bisphenol A	1 pM	9 pM	[61]	[62]
Ochratoxin A	0.3 pM	5 pM	[63]	[64]
Estradiol	1 fM	55 fM	[65]	[66]

**Table 3:** Selection of relevant small molecules measured in health monitoring. The current state-of-art testing method is mentioned, as well as whether an aptamer against the analyte is available or not.

Analyte	Indication	Current Testing Method	Aptamer Available? (Y/N)	Year	Ref.
Aldosterone	Hypertension	Radioimmunometric	N	-	-
Bilirubin	Hyperbilirubinemia	Colorimetric	N	-	-
Cortisol	Hypercortisolism/Hypocortisolism	Radioimmunometric	Y	2017	[71]
Creatinine	Kidney health	Fluorometric/Colorimetric	N	-	-
Estradiol	Hormonal illnesses in women	ELISA	Y	2007	[72]
Progesterone	Wide range of indications	Radioimmunometric	Y	2015	[73]
Testosterone	Wide range of indications	Radioimmunometric	Y	2017	[74]
Uric acid	Hyperuricemia/Hypouricemia	Fluorometric/Colorimetric	N	-	-
25(OH)D_3_	Vitamin D deficiency	HPLC	Y	2017	[33]
